# Feasibility of neural network metamodels for emulation and sensitivity analysis of radionuclide transport models

**DOI:** 10.1038/s41598-023-34089-9

**Published:** 2023-04-28

**Authors:** Jari Turunen, Tarmo Lipping

**Affiliations:** Tampere University, Pori, 28100 Finland

**Keywords:** Computational science, Mathematics and computing

## Abstract

In this paper we compare the outputs of neural network metamodels with numerical solutions of differential equation models in modeling cesium-137 transportation in sand. Convolutional neural networks (CNNs) were trained with differential equation simulation results. Training sets of various sizes (from 5120 to 163,840) were used. First order and total order Sobol methods were applied to both models in order to test the feasibility of neural network metamodels for sensitivity analysis of a radionuclide transport model. Convolutional neural networks were found to be capable of emulating the differential equation models with high accuracy when the training set size was 40,960 or higher. Neural network metamodels also gave similar results compared with the numerical solutions of the partial differential equation model in sensitivity analysis.

## Introduction

Since decommissioning of the Hanford plutonium production site in the U.S state of Washington, the site has been under an active monitoring and remediation program to reduce the risks of radioactive contamination of the surrounding area^1,2^. Nuclear liquid waste in the Hanford area was stored originally in buried single-wall steel tanks containing NaCl and NaOH solution^1^. This dangerous waste material is currently being transferred to newly-built double-wall tanks; plutonium production waste leakage into the ground and especially into the Columbia River is being monitored annually^3,4^.

When assessing the risk of contamination in Hanford, this leakage event can be compared to the Chernobyl incident in 1986, after which cesium (Cs) and strontium (Sr) radionuclides have been extensively studied. For example, doses of airborne ${^{137}}$Cs and ${^{90}}$Sr in food were monitored for over a decade in Romania. Peak values of 408.5 Bq/day of ${^{137}}$Cs and 1.48 Bq/day of ${^{90}}$Sr were measured in May 1986 from human food in^5^. Similar measurements were obtained in the Czech Republic where consumer milk was monitored for over a decade for ${^{137}}$Cs and ${^{90}}$Sr. Peak values of 506 Bq/l for ${^{137}}$Cs and of 0.1 Bq/l for ${^{90}}$Sr were obtained in 1986 in one of the samples^6^. In both cases, the ${^{137}}$Cs doses were more than two orders of magnitude greater than the ${^{90}}$Sr doses.

Despite the fact that the Chernobyl accident released both ${^{137}}$Cs and ${^{90}}$Sr^7^ and the nuclear waste in Hanford contains approximately 24% of ${^{137}}$Cs and 75% of ${^{90}}$Sr^1^, ${^{137}}$Cs is the major concern in many studies concerning radionuclide contamination. The main reasons for this might be that ${^{137}}$Cs bonds easily with chlorines to make salts, moves easily in the air, and dissolves easily in water^8^. ${^{90}}$Sr is not transported as easily as ${^{137}}$Cs, but plants growing in contaminated soil can take up small doses of both ${^{90}}$Sr and ${^{137}}$Cs^8,9^. In numerical simulations of groundwater, such as in^10,11^, ${^{90}}$Sr and ${^{137}}$Cs mainly decay in the transportation phase, while part of the ${^{90}}$Sr dose will stay in deep sediments and a fraction of both radionuclides could contaminate plants and animals. For example, ${^{137}}$Cs tends to be absorbed by mushrooms, while ${^{90}}$Sr tends to be spread to agricultural vegetables^11^.

Due to the easy dissolution of ${^{137}}$Cs in groundwater, the sediments in particular in Hanford may pose a threat to nearby residents. For that reason numerical simulations to determine the relation between transportation and absorption in sediments is essential. For example, Saiers et al. and Flury et al. have modeled the groundwater movement in Hanford sediments^12,13^. Both studies developed a partial differential equation (PDE) system fitted to the Hanford data. The results in both cases appear useful in making a numerical evaluation of cesium transportation and absorption in Hanford sediments.

Sometimes differential equation models are difficult to determine for some radionuclide interactions in the environment and very time consuming to solve. However, there are ways to address these problems. Reactive Transport Models (RTM) are one attempt to generalize sparse in-situ point data to cover the whole area to be investigated, while keeping the physical properties of the environment as accurate as possible. This can be done, for example, in the case of radionuclide transportation in groundwater. A good review of these types of complex models in the nuclear waste context is found in^14^. One of the earliest papers dealing with the usage of surrogate models to replace the original complex geochemical models is^15^. This paper showed that the Bayesian regularized neural network model performed almost equally as well as the original model. The importance of the training sample size selection is also discussed in^15^.

An alternative to RTMs are metamodels, based for example on Neural Network (NN) architectures. Neural networks are capable of approximating an arbitrary function provided that there are enough training data. The main idea behind metamodeling is that metamodels are capable of mimicking the behavior of the original model as accurately as possible with a much faster computation time than the original model.

When dealing with complex physical models, the uncertainty of the individual parameters and the sensitivity of the model output to its parameter values have been an active research area. Uncertainties may come from various sources such as the data being too sparse or obtained with faulty measurements, the model being inadequate for the purpose, and/or knowledge of the model parameters being too vague or, in the worst case, non-existent. One of the earliest examples of sensitivity analysis of biosphere models is^16^, where a simple aquatic ecosystem was modeled with random differential equations. The parameters were picked from Gaussian distributions with a known mean and standard deviation. After repeating this process numerous times, the distribution of the model output was obtained. In^17^ uncertainty is addressed using two different types of model for a coastal ecosystem. They modeled the system with two models: a compartment model and a dynamic-driven calibrated hydrodynamic model. Although the modeling approaches were different, the sensitivity analysis results obtained with the two models were similar.

Sensitivity analysis investigates the effect of the uncertainty or variability in the values of the model parameters on the model output. In^18^ five sensitivity analysis methods were compared in the context of a compartmental lake-farm biosphere model to study the sensitivity of the dose conversion factors from a spent nuclear fuel repository in bedrock to humans living in the area, to the parameters of the model such as distribution coefficients and concentration ratios between the various compartments. The results are presented in^19^. Both first and total order analyses were made and it was found that the order of the parameter sensitivities was similar for the different methods although the emphasis of the parameters varied. The sensitivity analysis of a model and its parameters is a time consuming task. A good review of the parameter reduction process with different sensitivity analysis methods is given in^20^.

Neural networks as metamodels have a significant benefit: when trained properly they are very fast in computation, making them attractive for sensitivity analysis. In^21^ a neural network metamodel has been used to simulate geological groundwater flow modeled using the Multiscale Finite Volume (MsFV) model. Neural networks were trained to learn MsFV basis functions with different resolutions. In the case of MsFV, the basis functions are computed using the matrix inverse operation, which is an operation that is demanding in terms of memory and computational power. In geological modeling, a coarse model is computed and refined using several small submodels. Submodel computing is also time consuming; however, when replacing the submodels with neural network metamodels, similar results were obtained as with the original model with significantly higher computational efficiency. Efficient computation was also one reason to suggest using a metamodel with sensitivity analysis in^21^. Three different metamodels (Deep Neural Networks, Gaussian Processes, and Polynomial Chaos Expansion) were tested against an RTM model in^22^. Their paper shows that the DNN model is a viable approach when modeling uncertainty propagation and performing Sobol’s first and total order sensitivity analyses.

In this paper we first numerically solve the Hanford sand PDE model presented in^13^. After that, we examine the possibility to find convolutional neural network metamodel architecture to mimic the PDE model. The idea in this approach is that the whole simulation surface can be computed in one computational phase. Subsequently, first and total order sensitivity analysis is performed using the Sobol method with both PDE and metamodel outputs.

## Methods

When assessing ${^{137}}$Cs transportation in groundwater in the Hanford case, the relationship between the free-water concentration and the pore-water concentration was determined in a laboratory experiment. The experiment was performed with Hanford sand packed in glass cylinders (length 7.2 cm and inner diameter 1.0 cm) using NaCl water solution to simulate the Hanford tank content. NaCl solution was pumped through the glass cylinders after which the ${^{133}}$Cs was injected into the tubes. The setup of the laboratory experiment is described in detail in^13^. Naturally, functions can be used to fit laboratory results data, thus, in the Hanford sand tube experiment case presented in^13^, the transport of ${^{133}}$Cs was described by the one-dimensional advection-dispersion equation (ADE) model:

1 $$\begin{aligned} {\theta \frac{\partial C}{\partial t}+\rho \frac{\partial S}{\partial t}=\theta D \frac{\partial ^2 C}{\partial z^2}-\theta V \frac{\partial C}{\partial z}}, \end{aligned}$$

where *C* is the solution-phase concentration, *S* is the sorbed-phase concentration, *t* is time, *z* is the distance, *h* is the volumetric water content, *q* is the bulk density, *D* is the dispersion coefficient, and *V* is the pore water velocity. This equation was extended with linear and nonlinear Freundlich isotherms in^13^. The two-site model is then:

2 $$\begin{aligned}{} & {} {S=S_1+S_2} \end{aligned}$$

3 $$\begin{aligned}{} & {} {S_1=fKC^n} \end{aligned}$$

4 $$\begin{aligned}{} & {} {\frac{\partial S_2}{\partial t}=\alpha [(1-f)(KC^n-S_2)]}, \end{aligned}$$

where $S_1$ and $S_2$ are the sorbed-phase concentrations for equilibrium and nonequilibrium sorption sites, *K* is the sorption coefficient, *n* is the Freundlich exponent, *f* is the fraction of equilibrium sorption sites, and $\alpha$ is the sorption rate coefficient.

The parameter values used in the experiment are listed in Table 1. The dispersion coefficient was not provided in^13^ and therefore the closest dispersion coefficient parameter value was selected from^23^ by comparing the candidate values with the other properties of the Hanford sand. Four parameters were subjected to sensitivity analysis with the tolerance limits presented in Table 2. Other parameters, such as saturated water content, sand bulk density, dispersion coefficient, and pore water velocity in Table 1 were omitted from the sensitivity analysis to follow the test setup described in^13^ as closely as possible.

The values of the four parameters in Table 2 were varied within their corresponding tolerance limits according to the Monte Carlo principle. Uniform distribution was used. The tolerances given in Table 2 were obtained from^13^ for NaCl solution concentrations of 100 mM and 1000 mM. As these tolerance limits are quite small, we also studied the effect of increasing the tolerance limits on the ability of the NN metamodel to learn the propagation dynamics of ${^{133}}$Cs in sand. For this purpose, tolerance limits of ${\pm }$ 50% around the mean value were used for the four parameters. These wider tolerance limits are given in Table 3 for the NaCl concentration of 100 mM. In the following, the tolerance limits of Tables 2 and 3 are referred to as narrow (NT) and wide (WT) tolerances, respectively.

**Table 1:** Parameter values used in the experiment.

Parameter	Symbol	Value	Unit
Time	t	0...0.20	h
Distance	z	0...7.20	cm
Volumetric (saturated) water content	${\theta }$	0.45	g/cm$^3$/cm$^3$
(Sand) bulk density	${\rho }$	1.54	g/cm$^3$
Dispersion coefficient	D	23.64	cm$^{2}$/h^23^
Pore water velocity	V	64.33	cm/h

Dispersion coefficient taken from^23^.

**Table 2:** Means and tolerances of the parameter values considered in sensitivity analysis.

Parameter	Symbol	Value (100 mM)	Value (1000 mM)	Unit
Sorption rate coefficient	${\alpha }$	0.163 ${\pm }$ 0.011	0.115 ${\pm }$ 0.001	1/h
Fraction of equilibrium sorption sites	*f*	0.339 ${\pm }$ 0.023	0.324 ${\pm }$ 0.001	
Sorption coefficient	*K*	31.71 ${\pm }$ 1.02	3.14 ${\pm }$ 0.042	ml/g
Freundlich exponent	*n*	0.810 ${\pm }$ 0.015	0.76 ${\pm }$ 0.001

**Table 3:** Means and wider tolerances of the parameter values considered in second sensitivity analysis.

Parameter	Symbol	Value (100 mM)	Unit
Sorption rate coefficient	${\alpha }$	0.163 ${\pm }$ 0.0815	1/h
Fraction of equilibrium sorption sites	*f*	0.339 ${\pm }$ 0.1695	
Sorption coefficient	*K*	31.71 ${\pm }$ 15.855	ml/g
Freundlich exponent	*n*	0.810 ${\pm }$ 0.405	

**Figure 1:**
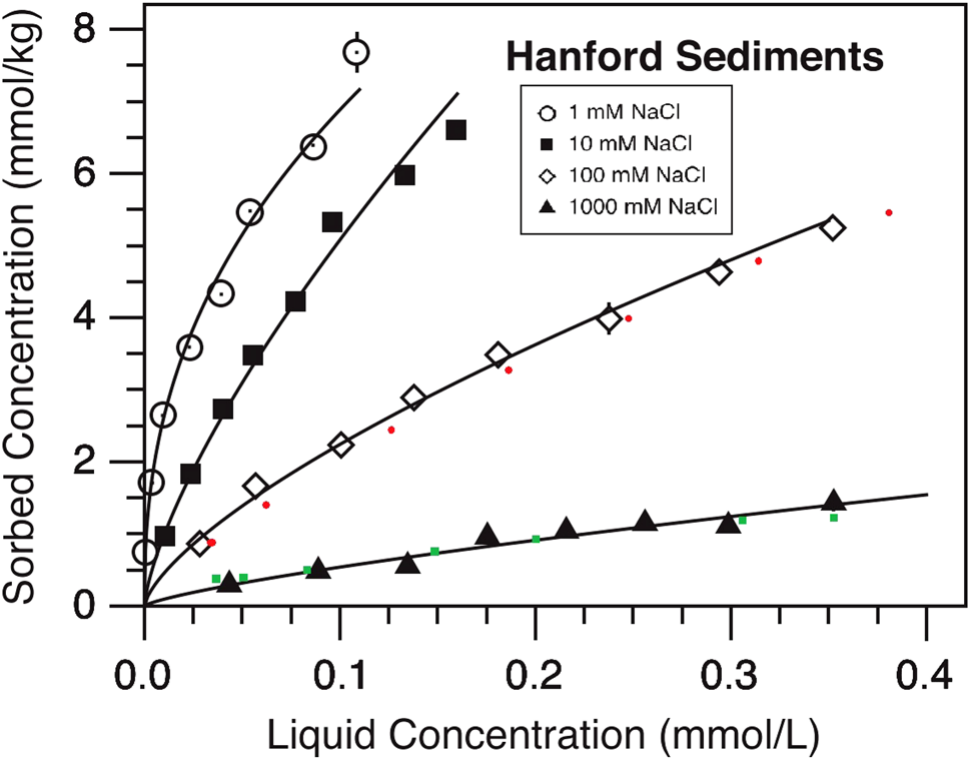
Liquid versus sorbed concentrations after reaching the equilibrium state. The figure is modified from^13^. The small circles, triangles, and squares indicate the original experimental test results, the curves indicate the results of the PDE models presented in^13^ while the red and green dots indicate the 100 mM and 1000 mM NaCl solution results, respectively, obtained using the *FiPy* package.

The Hanford model was numerically solved using the Python *FiPy* package^24^. The relationship between the reference values from^13^ and the computed values is shown in Fig. 1. The mean values of parameters ${\alpha }$, *f*, *K*, and *n* from Table 2 were fed into the Python code and the PDE (ADE complemented by both Freundlich isotherms) was solved for both the 100 mM and 1000 mM NaCl solutions. The final combinations of the outputs, liquid (C) versus sorbed (S) concentrations, are shown in Fig. 1. The green and red dots (corresponding to 1000 mM and 100 mM NaCl solutions, respectively) were obtained by simulating the injection of ${^{133}}$Cs in concentrations between 0.1 and 1.3 mM. The model parameters were available only for 100 mM and 1000 mM NaCl solution of the Hanford sand in^13^. It can be seen from the figure that, in the case of 100 mM NaCl solution, there is a minor offset between the experimental results from^13^ and the numerical solution of the PDE calculated using the *FiPy* package. It should be noted that the original curves in Fig. 1 are empirical fittings for the data points obtained in experiments in^13^, and the red dots are still within the tolerance limits for the experimental results presented in^13^. One reason for the slight bias might be the missing dispersion coefficient that was replaced using values from^23^. When comparing the 100 mM and 1000 mM solution results, the parameters may emphasize the effect of dispersion coefficient more in the 100mM solution than in the 1000 mM solution, causing the minor deviation of the red dots from the empirically fitted line in Fig. 1.

**Figure 2:**
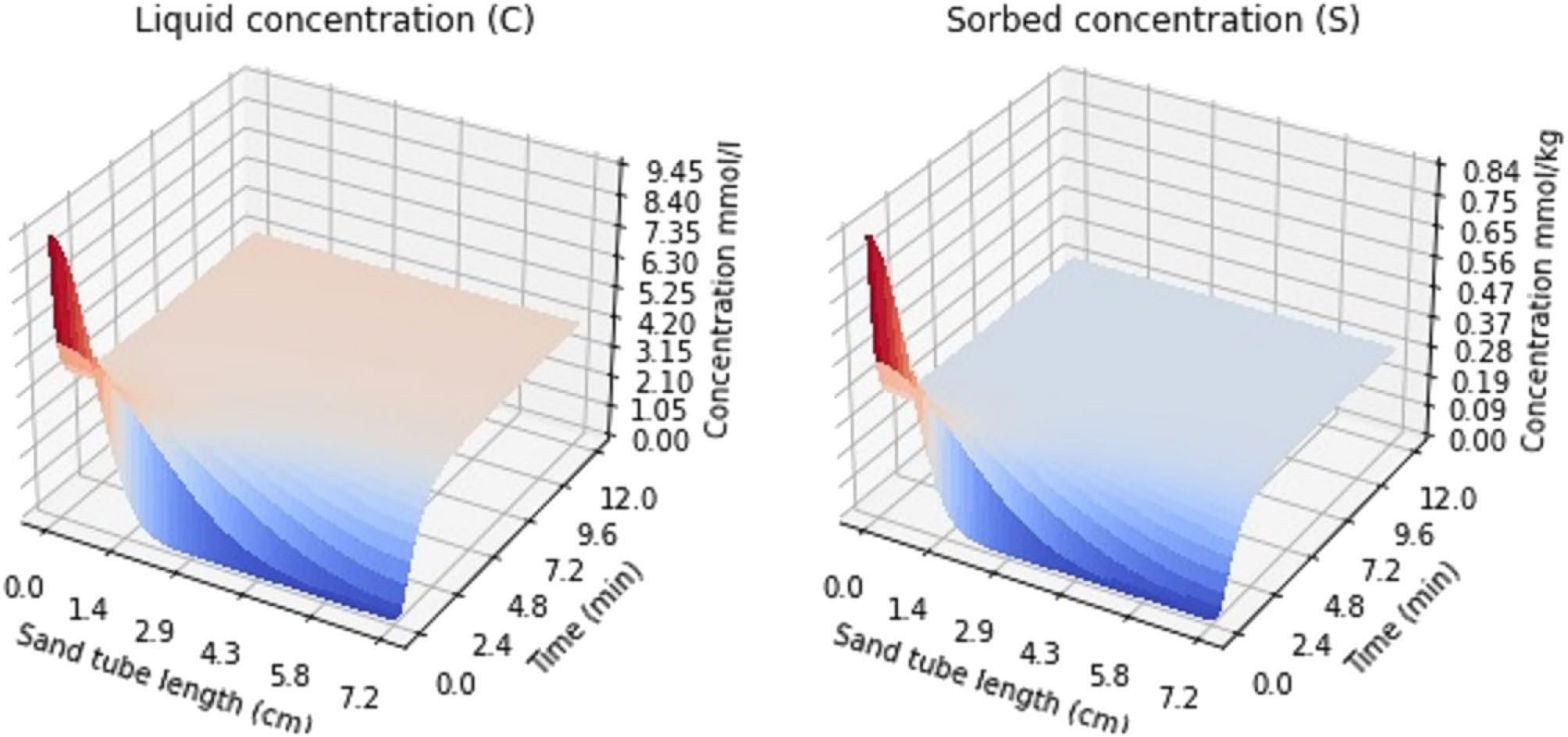
Sorbed and liquid concentrations obtained by numerically solving the PDE using the *FiPy* package. The parameter values used were: ${\alpha }$ = 0.163, *f* = 0.339, *K* = 31.71, and *n* = 0.810. The simulations concern 1 mM Cs injection at the time instant 0.0 s.

The 100 mM NaCl solution PDE model was selected for further consideration. The PDE output was computed using the *FiPy* package one time instant at a time, producing a one-dimensional concentration curve mimicking the glass tube experiment in^13^. However, these time instants can be stacked side by side according to the distance from the injection point of the ${^{133}}$Cs. The PDE solution thus forms a surface, as presented in Fig. 2. The initial Cs concentration at time step 0 was 1 mM in all the simulations. It can be seen from Fig. 2 that the ${^{133}}$Cs gradually spreads along the tube and eventually reaches equilibrium state. These 100 mM NaCl solution equilibrium state values at 12 min from the injection time for liquid and sorbed concentrations are represented by the red dots in Fig. 1.

**Figure 3:**
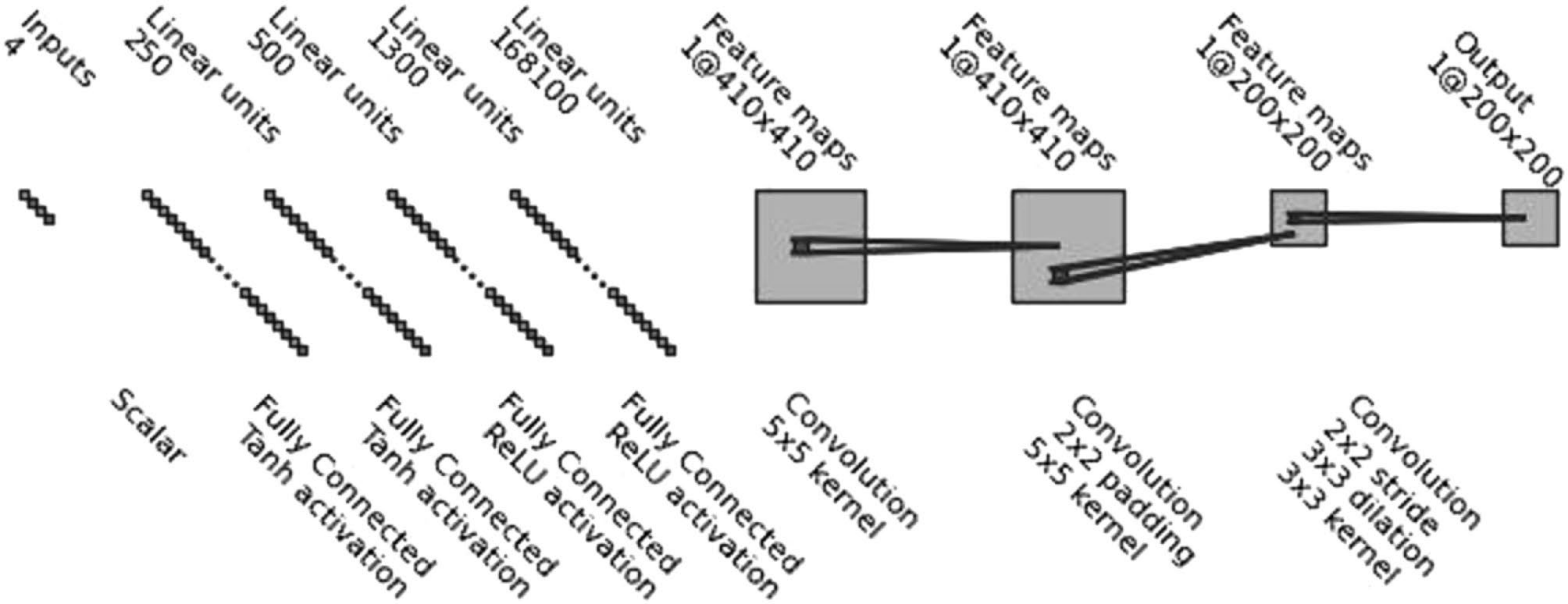
Proposed neural network architecture to model the concentration surfaces and perform sensitivity analysis of the four parameters presented in Table 2. The first convolution layer is followed by batch normalization and the second by batch normalization and ReLU operations before the data are fed to the third convolutional layer. (The figure was generated by adapting the code from https://github.com/gwding/draw_convnet).

After successful verification of the PDE model, a neural network metamodel was designed. The idea in this proof-of-concept network design was to obtain the whole Hanford sand concentration surface (i.e., time vs distance in the tube) at once. The network architecture was designed and implemented using the Python *pytorch* package. After a long optimization process where the Bayesian search methods from the Python *scikit*-*optimize* package were used, the neural network architecture presented in Fig. 3 was found to show good results. In the evaluation of model architectures, narrow parameter tolerances of Table 2 were used. However, due to the high computer memory demand to keep the training data, the models of concentration surfaces for liquid concentration (C) and sorbed concentration (S) had to be trained separately. The network architecture presented in Fig. 3 was used in all the experiments. The networks were trained using 120 epochs with an initial learning rate of 0.005 with automatic reduction. 90% of the training data were used for training and 10% for model validation. Test data were produced for further analysis after the training of the models had been done. The network architecture was kept the same in the experiments; only the training set size was varied between 5120 and 163,840.

The CNN model architecture was cloned and trained using several data sets to determine the required amount of data for emulation of the PDE model. The mean square error (MSE) between the PDE solution (computed using the *FiPy* package) and the CNN output was used as the error criterion in network training. Networks trained with different data set sizes and their respective MSE errors using test data are presented in Table 4.

**Table 4:** MSE error with training sets of different size.

Number of realizations used for network training	C (MSE/sample) using test set of size 10,240	S (MSE/sample) using test set of size 10,240
5120	16.423e−5	< 1e−8
10,240	1.910e−5	< 1e−8
20,480	0.313e−5	< 1e−8
40,960	0.155e−5	< 1e−8
81,920	0.153e−5	< 1e−8
163,840	0.145e−5	< 1e−8

Narrow parameter tolerances are used.

Both the concentration surfaces obtained by numerical solution of the PDE using the *FiPy* package (hereafter: DE surfaces) and the surfaces obtained by the CNN metamodel (hereafter: NN surfaces) were then analyzed using the Sobol first and total order sensitivity analysis methods using the Python *SAlib* package. In the Sobol method, the dimensionality is increased by decomposing the output variance of the model into summands of input parameter variances^25,26^. In first order analysis these summands are divided by the total sum of variances, and the outcome is the individual parameter sensitivity index averaged over variations in other input parameters^25^. In Sobol’s total order sensitivity analysis, the summand variances include all interaction variances between the parameters divided by the total sum of variances^25^.

## Results

**Figure 4:**
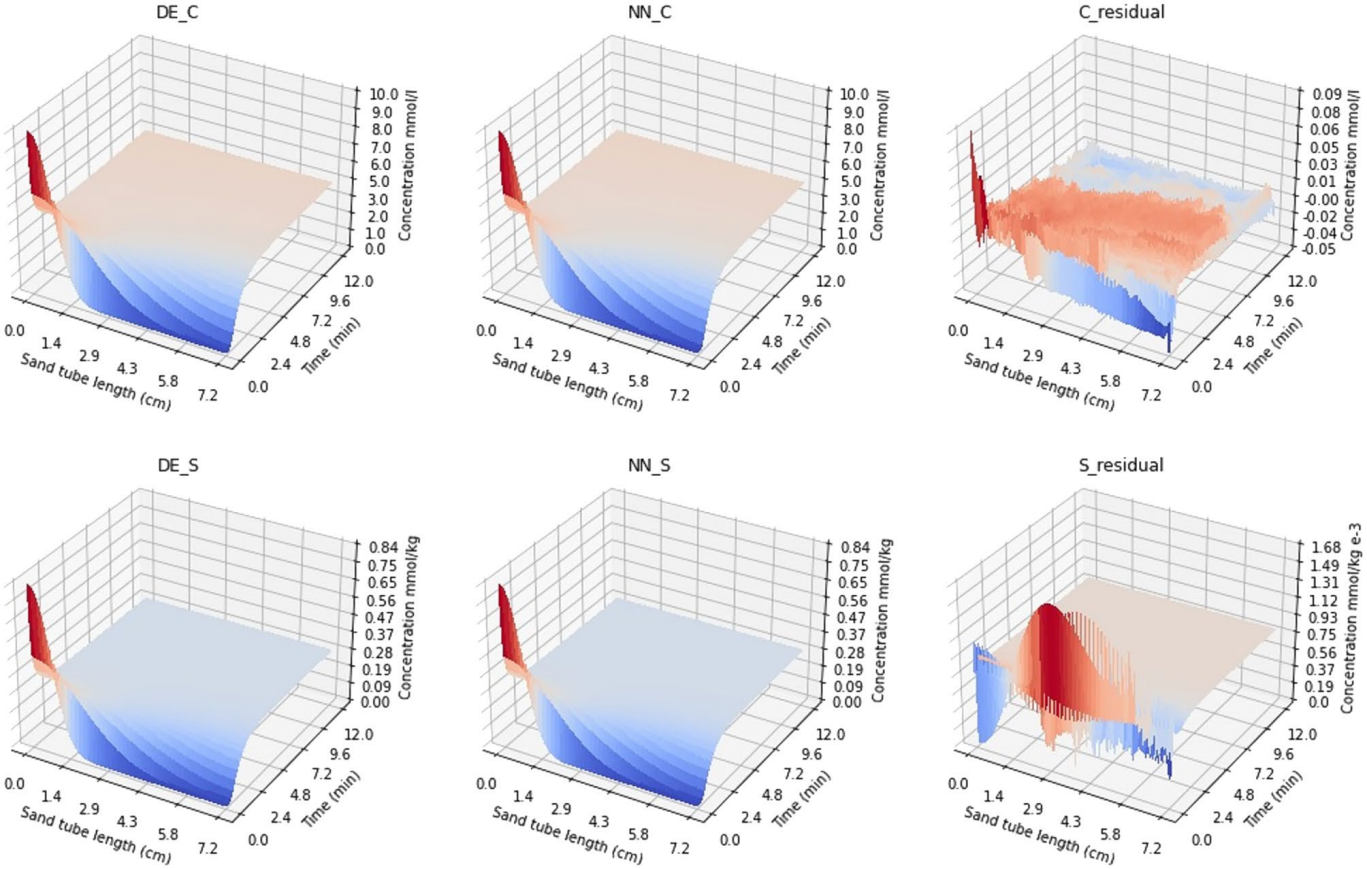
Comparison between DE and NN (trained using 163,840 realizations) concentration surfaces for both liquid (C) and sorbed (S) concentrations. The results are presented for mean parameter values ${\alpha }$ = 0.163, *f* = 0.339, *K* = 31.71, and *n* = 0.810.

A comparison between the DE and NN concentration surfaces is shown in Fig. 4 when the CNN was trained using 163,840 realizations for both liquid and sorbed concentrations. It can be seen that the error is largest at the beginning ($t < 1$ s) of the simulations, being at most of the order of 0.1 mmol/l for C and 0.001 mmol/kg for S. This is at least two orders of magnitude less than the concentration values.

**Figure 5:**
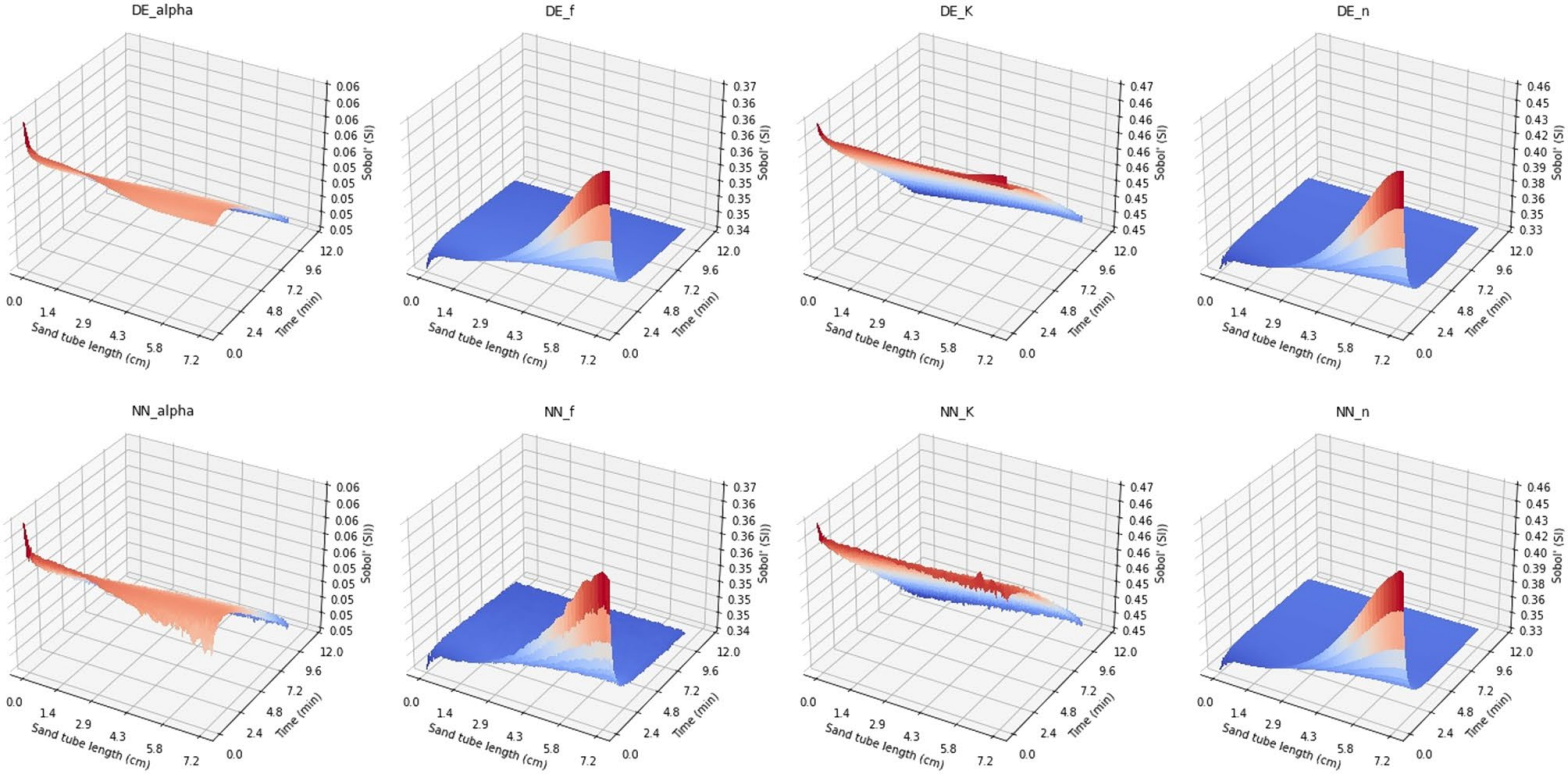
Sobol’s first order sensitivity analysis results for narrow tolerances for DE and 40,960-trained NN concentration surfaces for liquid concentration C.

**Figure 6:**
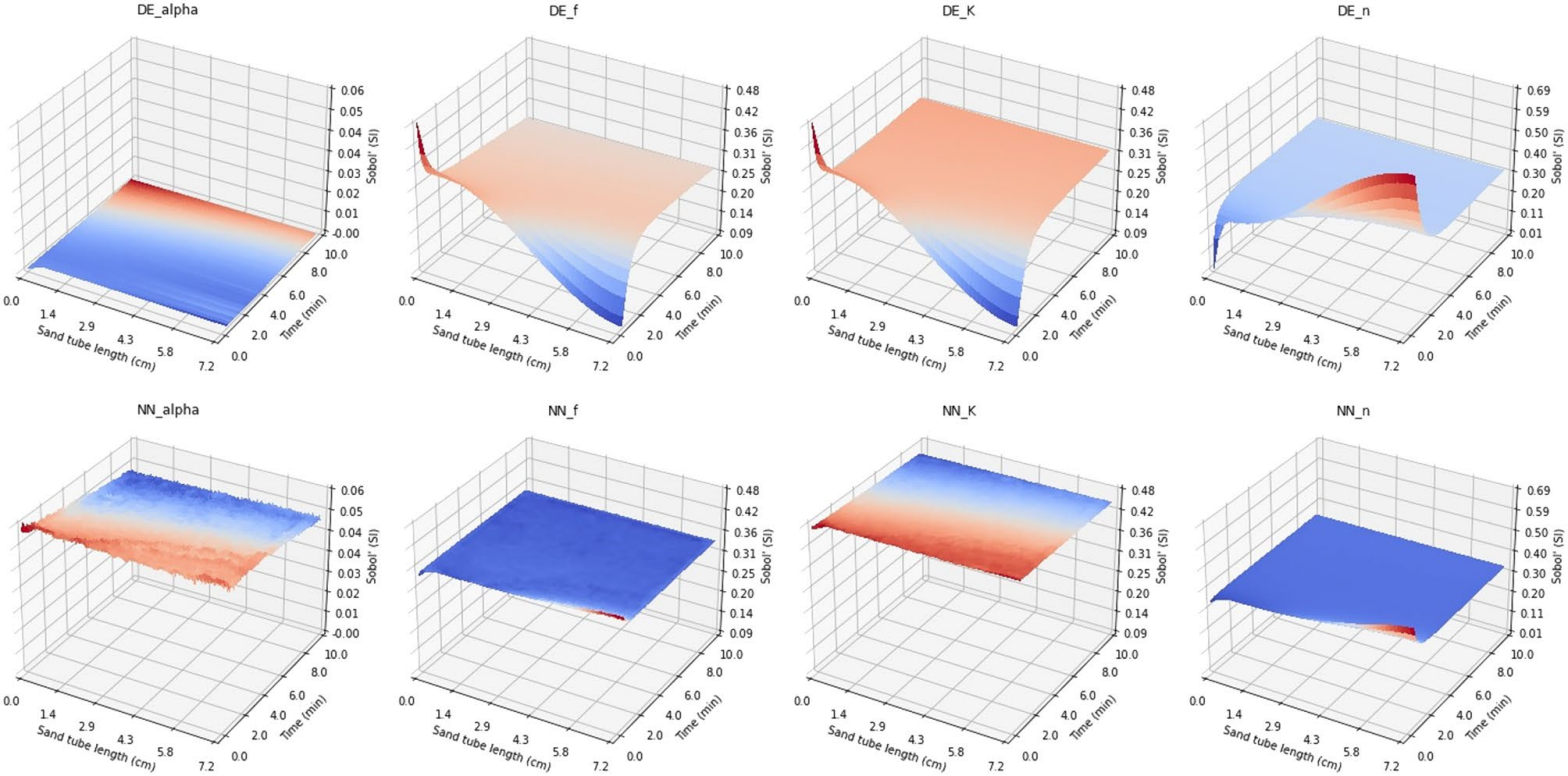
Sobol’s first order sensitivity analysis results for wide tolerances for DE and 40,960-trained NN concentration surfaces for liquid concentration C. The architecture was kept the same.

10,240 DE and NN surfaces were then stacked on a pixel-by-pixel basis for sensitivity analysis using Sobol’s first and total order analysis methods. The CNN trained using 163,840 realizations (with narrow parameter tolerances) was selected for the sensitivity analysis. The results for Sobol’s first order sensitivity analysis for the liquid concentration case using narrow and wide parameter tolerances are presented in Figs. 5 and 6, respectively. The sorbed concentration case was omitted at this point as varying the parameter values within the tolerance limits of either Table 2 (NT) or Table 3 (WT) yielded virtually the same concentration surface for the DE model. As the NN model was trained using the DE model results, the NN model gave also the same sorbed concentration values and the error between the two was negligible (see Table 4). One reason for this might be that constant initial cesium concentration value of 1 mM was used.

**Figure 7:**
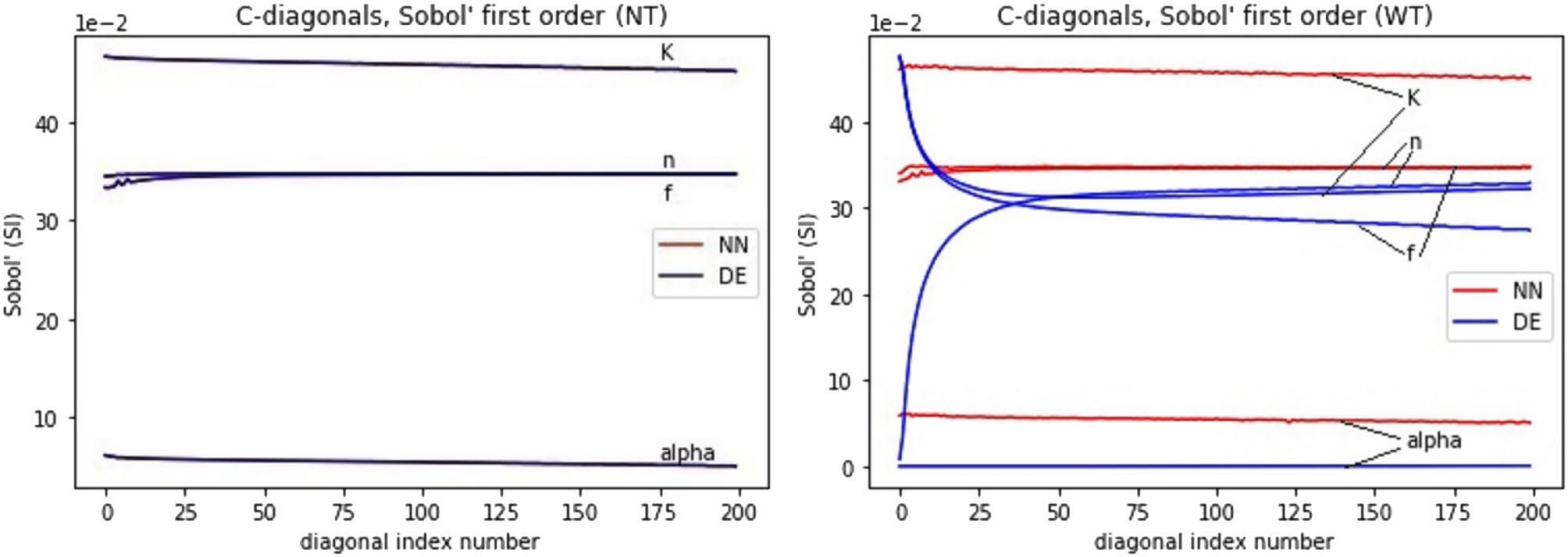
First order sensitivity analysis results along the diagonal of the time-distance plane of the model solution for the four parameters considered in the sensitivity analysis. CNN metamodel is trained using 40,960 realizations with narrow parameter tolerances. The left and right panels show the sensitivity analysis results for narrow and wide parameter tolerances, respectively.

It can be seen from Fig. 5 that both models, DE and NN, give almost identical results using Sobol’s first order analysis with narrow tolerances. The sensitivity analysis results obtained using the NN metamodel have somewhat higher variance; however, the main trends along time and the distance within the test tube are very similar to those obtained with the DE model. Also, the relative sensitivity to the four parameters is similar for the two models. Figure 7, presenting the first order sensitivity index values along the diagonal of the time-distance plane (starting from (0 cm, 0 min) to (7.2 cm, 12 min) in 200 steps), shows that the Sobol method gives most weight to parameter *K*. Parameter *K* is a multiplier (as well as *f*) in Eqs. (3) and (4) so it seems plausible that the model’s sensitivity to this is greater than to the other parameters. The difference between *K* and *f* can be seen in Eq. (4) where *K* is multiplier of $C^n$ and *f* is inverse multiplier of $(KC^n-S_2)$. Also, the model is least sensitive to the ${\alpha }$ parameter in the first order Sobol’s sensitivity analysis; it is a multiplier in Eq. (4) and its tolerance is quite small when compared to *K* and *f* in Table 2. In addition, *K* and *f* appear in Eqs. (3) and (4), whereas ${\alpha }$ only appears in Eq. (4). It should be noted that in Figs. 5 and 6 the sum of the individual pixels in the DE and NN surfaces is not equal to one. This is probably explained by Eq. (3) where the *K* and *f* parameters are multiplied causing interaction between the two parameters.

When comparing Figs. 5 and 6 it can be seen that when using wider parameter tolerances in first order sensitivity analysis, the behavior of the sensitivity index values along the time-distance plane changes for both models. Also, the sensitivity index surfaces for the DE and NN models diverge with wider tolerances. In Fig. 7 the ranking order of model sensitivity to the four parameters is illustrated. While the NN metamodel is not sensitive to the parameter tolerances, giving similar results for the WT case as for the NT case, the sensitivity indices obtained for the DE model change when wider tolerances are used. The sensitivity to the $\alpha$ parameter drops to zero and the sensitivity to the *K* parameter drops to the level close to that for the *f* and *n* parameters. It should be noted that the tolerances of Table 2 (i.e., narrow tolerances) were obtained from^13^ and are therefore more realistic.

**Figure 8:**
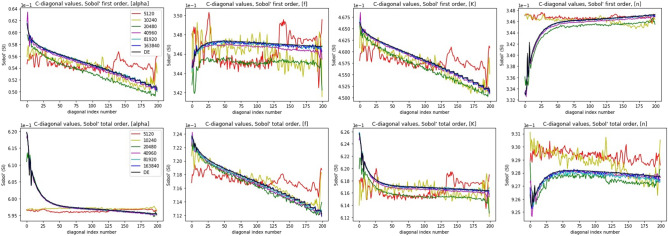
Sensitivity analysis results along the diagonal of the time-distance plane of the model solution for Sobol’s first (upper row) and total (lower row) order methods. The *FiPy* packet solution as well as the CNN metamodel results using training sets of various sizes are presented. Sensitivity analysis is performed using 10,240 realizations with narrow parameter tolerances.

**Figure 9:**
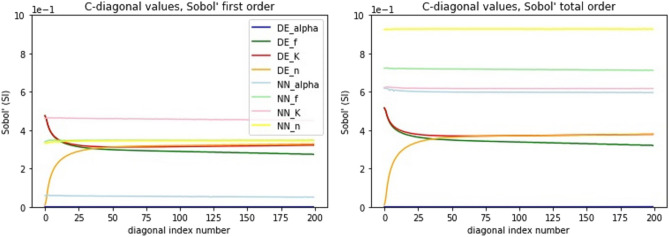
Sensitivity analysis results along the diagonal of the time-distance plane of the model solution for Sobol’s first and total order methods using wider tolerances. The *FiPy* packet solution as well as the results for the 40,960-trained CNN metamodel are presented.

In Fig. 8 the sensitivity analysis results for first and total order Sobol methods are compared using narrow parameter tolerances. The sensitivity index values along the time-length plane are presented for the DE model and the CNN metamodels trained with different number of realizations. The results indicate that the sensitivity index values obtained with the CNN metamodel virtually coincide with those obtained with the DE model if the training set size is above 40,960. Even for training set size as low as 5120, the baseline sensitivity index values obtained with the metamodel are similar to those obtained with the DE model (note the narrow range of the vertical axes). The lower row of panels in Fig. 8 shows that switching to the total order Sobol method gives substantially different sensitivities to the four parameters for both DE and NN models. The results for the DE and NN models coincide also for the total order analysis though.

Finally, in Fig. 9 the sensitivity index values along the time-length plane are given for the first (left panel) and total (right panel) order Sobol analyses using the wider parameter tolerances. The left panel here contains the same information as the right panel of Fig. 7. When looking at the right panel of Fig. 9 and comparing it to the lower row of panels in Fig. 8 shows that using wider tolerances for the parameters preserves the relative sensitivities in the NN model case but not in the DE model case (note the different scales of the vertical axes in the left and right panels). On the other hand, comparing the left and right panels of Fig. 9 shows that switching from the first order to total order Sobol’s sensitivity analysis method preserves the sensitivities of the DE model but not those of the NN model to the four parameters.

## Discussion

The first aim of this paper was to determine whether convolutional neural network metamodels are feasible for simulating the transport of radionuclides in sediments. The experiment described in^13^ and modeled by the PDE of Eqs. (1)–(4) was considered as a test case. The CNN metamodel was found to be a promising alternative to the original PDE model. In terms of computation time, solving the PDE system using the *FiPy* package for 10,240 realizations over the 200 $\times$ 200 grid covering the time-distance plane of (0 min, 0 cm) to (12 min, 7.2 cm) took 235 min (parallel 16-core PC), while the same calculations using a neural network metamodel took only 3.5 min (GPU assisted). This is a remarkable saving of computation time.

However, there are several issues when designing and training a neural network based metamodel. While the computation of the neural network metamodel is fast, even with this simple experiment it took a great deal of time (months) to find a decent architecture for the model. However, in the future when more experience and insight into the problem will been obtained, finding a well-performing network architecture will require less experimentation.

Also, the number of training samples is a concern in many cases where neural network metamodels are used. In the experiment described in this paper the error between the PDE solution and the metamodel result was considered sufficiently low when a training set of 40,960 realizations was used. It was noted, however, that the metamodel was feasible only for the liquid concentration case, whereas numerical instability problems were faced when solving for the sorbed concentration.

One of the reasons to use metamodels is the analysis of the sensitivity of the model to its parameters. Therefore, our second aim was to find whether a neural network metamodel can, in fact, be used for the sensitivity analysis in the Hanford case. The following aspect were addressed:

- how many realizations should be used for training the NN metamodel to obtain similar sensitivity indices as with the DE model?
- how do the tolerances of the model parameters used in sensitivity analysis affect the sensitivity index values obtained with the DE and NN models?
- how do the sensitivity indices obtained with the first and total order Sobol sensitivity analysis methods compare for the DE and NN models?

In all the experiments performed to answer these questions the NN metamodel was trained using the tolerances of the parameter values given in Table 2 (i.e., narrow tolerances)^13^. Also, in all sensitivity analysis calculations, 10,240 realizations were used.

When the number of realizations used for training the NN metamodel was 40,960 or more, the sensitivity indices obtained with the metamodel followed closely those obtained with the DE model (see Fig. 8). Using less realizations for network training still gave similar sensitivity values for the two models, however, the variability of the values for the NN metamodel was higher. The results indicate that NN metamodels can emulate respective DE models well also for the purpose of sensitivity analysis.

For the calculations performed to address the other two questions above, the 40,960-trained base NN metamodel was used. When comparing the sensitivity indices obtained with the DE and NN models using narrow vs wide parameter tolerances and first vs total order Sobol methods, interesting results emerged. The NN metamodels appeared insensitive to the parameter tolerances in both first and total order analysis. However, the first and total order Sobol analysis yielded substantially different sensitivity index values. The sensitivity index values obtained with the DE model were different for the narrow and wide parameter tolerances as also for the first and total order Sobol analysis in the NT case. However, when the parameter tolerances were increased, the sensitivity indices obtained with the DE model where similar for the first and total order analysis.

Although sensitivity analysis and quantification of uncertainties become more widely used and feasible with the increase in available computational power, the understanding of how model properties affect the results of the various sensitivity analysis methods is still vague. The situation becomes even more complex when metamodels are used to emulate PDE models. As seen from our study, although the DE model and NN metamodel gave similar concentration surfaces, they behaved in a different manner when sensitivity analysis was performed.

Physics Informed Neural Networks (PINNs) would be an ideal solution in these types of problems, where the model is relatively simple and the differential equations can be defined. PINN training can be done simultaneously when computing the DE, thus saving time. In addition, during training, PINNs utilize the governing differential equations in the learning process with sparse data^27^. A few Python packages are available for PINN network training. For example, in^28,29^ the physical problem is first defined in the form of ordinary differential equations (ODE) or partial differential equations (PDEs). Then the network architecture is defined and the network is trained at the same time as the ODE/PDE is solved by feeding the ODE/PDE derivatives to the network during the training process. Unfortunately, the Python based open source PINN architectures are restricted to basic neural network components at the moment, restricting the scope of problems that could be solved with them. PINNs are an attractive modeling target for future research, by changing the architecture into one that is suitable for PINNs. However, the aim in this paper was to find the feasibility of solving the whole concentration surface at once. In spite of the quick computation of convolutional neural networks using GPU assistance, it is very memory consuming and changes in the architecture are difficult to perform. Therefore the PINN approach could be quite attractive for this type of problem.

Ordinary and deep learning networks are more flexible and trainable when there are enough data (i.e., input-output pairs) available for the problem at hand. The question is, how much time and effort one wants to put in investigating various network architectures to determine the optimal solution. Also, new architectures are being developed and recently better results have been obtained using transformer networks in a variety of application areas. It is likely that the design and development time of neural network architectures will be reduced in the future when computational power increases.

## Data Availability

The data sets used and analyzed during the current study are available from the corresponding author on reasonable request.
